# Metal Nanodot Array via Thin Oil Layer‐Assisted Dropwise Solid‐State Dewetting

**DOI:** 10.1002/smsc.202500311

**Published:** 2025-09-11

**Authors:** Hyesun Hwang, Jihye Kim, Seungbae Jeon, Seong‐Min Jo, Sungmin Park, Hyosung An, Michael Kappl, Hans‐Jürgen Butt, Sanghyuk Wooh

**Affiliations:** ^1^ Department of Chemical Engineering Chung‐Ang University 84 Heukseok‐ro Dongjak‐ju Seoul 06974 Republic of Korea; ^2^ Advanced Materials Division Korea Research Institute of Chemical Technology 141 Gajeong‐ro Yuseong‐gu Daejeon 34114 Republic of Korea; ^3^ Department of Biomaterial Science Pusan National University 1268‐50, Samrangjin‐ro, Samrangjin‐eup Miryang‐si Gyeongsangnam‐do 50463 Republic of Korea; ^4^ Physical Chemistry of Polymers Max Planck Institute for Polymer Research Ackermannweg 10 55128 Mainz Germany; ^5^ Department of Petrochemical Materials Engineering Chonnam National University 19 Samdong 3‐gil Yeosu‐si Jeollanam‐do 59631 Republic of Korea; ^6^ Physics at Interfaces Max Planck Institute for Polymer Research Ackermannweg 10 55128 Mainz Germany

**Keywords:** metal deposition, metal nanodot array, solid‐state dewetting, surface energy, thin oil layer

## Abstract

Precise control of metal nanodot arrays is crucial for optimizing their plasmonic, catalytic, and photonic properties. Fabrication methods for homogeneous nanodots generally rely on complex processes, such as lithography or layer‐by‐layer assembly. Recently, nanodot array fabrication via metal deposition, e.g., sputtering and thermal evaporation, has received attention due to its simplicity and scalability. However, structures produced by deposition are often inhomogeneous and suffer from size limitations, because metals generally possess high surface energy in air. In this study, we propose a strategy for fabricating uniform metal nanodot arrays through thin oil layer‐assisted solid‐state dewetting. Metals are deposited by sputtering onto a glass substrate coated with a thin oil layer, followed by thermal annealing that induces dewetting and the formation of nanodot. Surfactants incorporated in the oil reduce the surface energy of metals, thereby suppressing undesired coalescence. Additionally, the size and shape uniformity of the resulting nanodots are improved and can be controlled by adjusting deposition thickness and/or oil layer thickness. This simple strategy, based on surface stabilization using oil/surfactant, effectively overcomes the limitations of deposition methods. Furthermore, nanodot arrays composed of various metals, or two or more metals, can be fabricated, providing a versatile and customizable platform for nanostructure engineering.

## Graphical abstract

A strategy is introduced to fabricate homogeneous metal nanodot arrays through solid‐state dewetting assisted by a thin oil/surfactant layer. The thin oil/surfactant layer effectively helps to reduce and stabilize the surface energy of metal, leading to controlled dewetting. This approach offers a customizable route to control nanostructure morphology and optical properties.

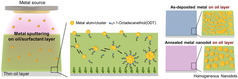

## 1 Introduction

Nanostructured metals have been explored for a wide range of research and applications in modern science and engineering.^[^ ^1^, ^2^, ^3^, ^4^ ^]^ Metal nanodot arrays formed on substrates are a representative structure for utilizing nanoscale metals. Depending on the composition, size, shape, and density of the nanodots, unique and tunable functionalities can be achieved.^[^ ^5^, ^6^, ^7^, ^8^ ^]^ Consequently, numerous methods to produce nanodot arrays have been developed over the past few decades, including bottom‐up chemical synthesis,^[^ ^9^, ^10^ ^]^ template‐assisted growth,^[^ ^11^, ^12^ ^]^ and top‐down lithography,^[^ ^13^, ^14^, ^15^ ^]^ offering control over particle size, distribution, and composition. Although these approaches provide high precision and scalability, they often require multi‐step fabrication processes, specialized equipment, or lithographic patterning. Among the available techniques, metal deposition methods, by sputtering or thermal evaporation, have gained attention due to their material versatility and compatibility with large‐scale production without relying on prepatterned templates.^[^ ^16^, ^17^, ^18^, ^19^ ^]^ In this approach, metal grains initially form film‐like continuous structures upon deposition. Subsequent thermal annealing induces solid‐state dewetting, leading to the generation of isolated metal nanodots.^[^ ^20^, ^21^, ^22^, ^23^ ^]^

However, until now, metal nanodots produced by deposition‐based methods have typically exhibited irregular shapes and inhomogeneous sizes.^[^ ^24^, ^25^ ^]^ Controlling both size and morphology is challenging because metals have high surface energies, which promote undesired coalescence and morphological changes of nanodots in air.^[^ ^26^ ^]^ Moreover, limited deposition thickness presents an additional challenge. Deposited metals on a solid substrate undergo breakup into discrete nanodots or island‐like structures during thermal annealing via solid‐state dewetting, driven by the minimization of surface energy.^[^ ^27^, ^28^ ^]^ In the case of ultrathin metal layers (<5 nm), dewetting occurs more easily due to the high density of grain boundaries.^[^ ^29^, ^30^, ^31^ ^]^ In principle, the amount of deposited metal serves as a key parameter to control nanodot size. However, as the metal thickness increases, coalescence becomes dominant, resulting in randomly interconnected domains rather than fragmentation into isolated nanodots.^[^ ^32^, ^33^ ^]^ Therefore, the nanodot size that can be controlled by metal deposition is limited.

Indeed, most issues associated with deposition‐based nanodot fabrication originate from the high surface energy of metals. Alternative strategies by deposition for reducing surface energy using surfactant‐mediated or structure‐guided dewetting methods have also been explored.^[^ ^34^, ^35^ ^]^ However, these approaches often involve complex steps or additional substrate architecture. To address these limitations, in this study, we propose a strategy to reduce the surface energy of deposited metals that facilitates solid‐state dewetting while suppressing uncontrolled coalescence. Here, we introduce a method that utilizes a thin oil layer on the top surface of the substrate. The oil itself lowers the surface energy of the deposited metals, which helps to promote homogeneous size distribution and fabrication of nanodots over a broader size range. In addition, the oil layer acts as a reservoir medium for surfactants.^[^ ^36^ ^]^ Surfactants embedded in the oil further stabilize the metal nanodot surfaces, which improves size uniformity and shape regularity.^[^ ^37^, ^38^ ^]^ Notably, this approach enables the formation of larger nanodots even with thick metal deposition, as the presence of oil and surfactant facilitates solid‐state dewetting easily. Moreover, the method supports the fabrication of bi‐ or multi‐component nanodot arrays via sequential metal deposition. It offers compositional versatility and tunable optical properties, providing a platform for metal nanodot applications in plasmonic, catalysis, and optoelectronics.^[^ ^39^, ^40^, ^41^ ^]^

## 2 Results and Discussion

**Figure 1:**
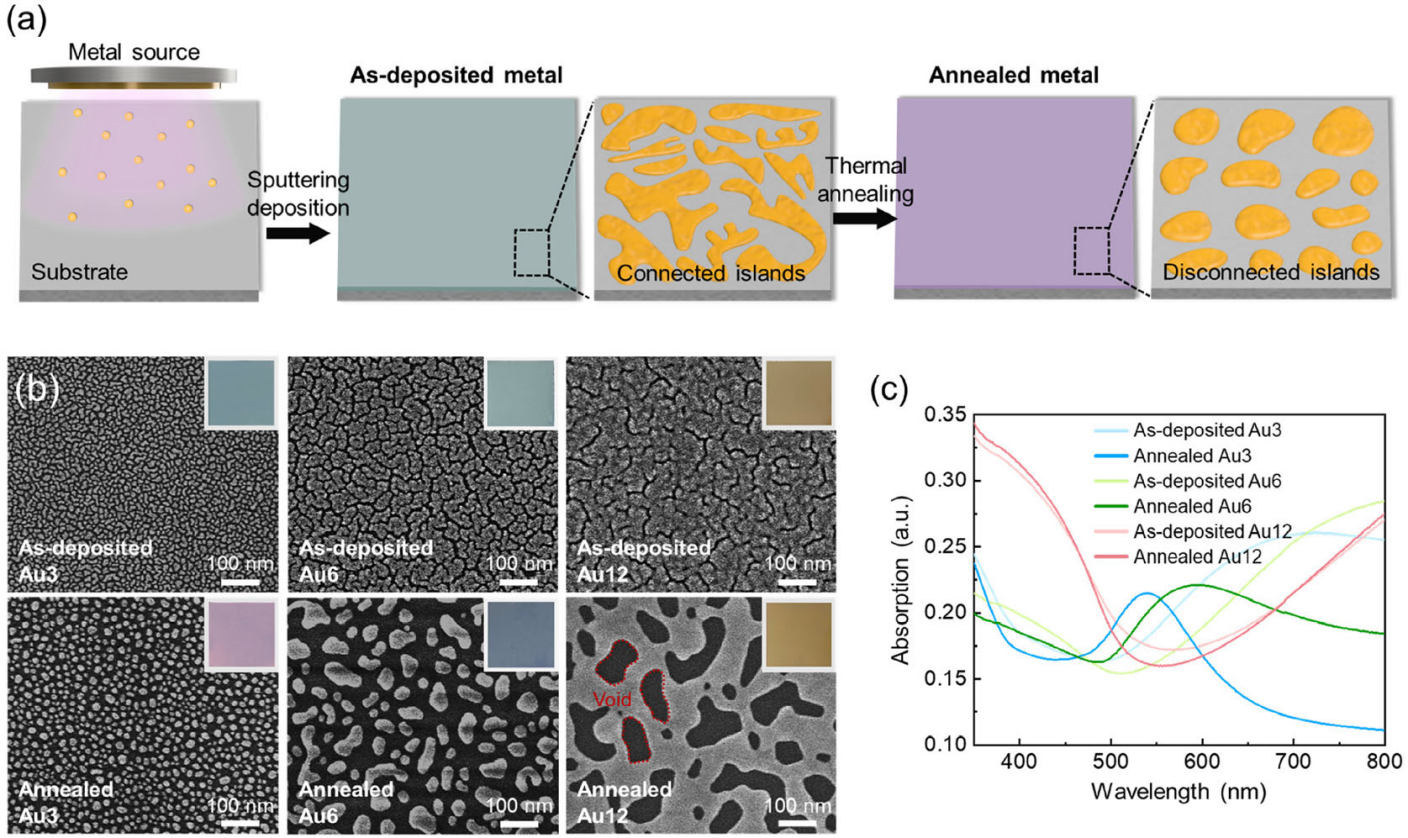
a) Schematic illustration of generation of metal nanodot array on flat substrate by metal deposition followed by thermal annealing. b) Morphology of the deposited gold (Au) on the substrate with varying deposition thicknesses (3 nm (Au3), 6 nm (Au6), and 12 nm (Au12)) and after annealing. Inset images are the corresponding optical appearance of substrates. c) UV–vis absorption spectra of as‐deposited and annealed Au. The annealing temperature is 250 °C for 120 min.

We present a method to generate metal nanodot arrays on flat substrates through metal deposition followed by thermal annealing (**Figure** **1**a). Thin metal layers are physically deposited onto the substrate by sputtering or thermal evaporation, initially forming connected island‐like structures. Then, annealing induces solid‐state dewetting, leading to the disconnection of these structures and the formation of isolated nanodots. This transformation occurs even at temperatures below the melting point of metals, as the system tends to minimize surface energy due to the inherently high surface energy of metals, exceeding several hundred mN m^−1^.^[^ ^42^ ^]^ For example, deposited gold (Au) layers of varying thicknesses on the substrate show mostly connected island structures prior to annealing (Figure 1b). Here, polydimethylsiloxane (PDMS) grafted hydrophobic glass is selected as the substrate to introduce the oil layer.^[^ ^43^, ^44^ ^]^ Before applying the oil layer, the 3 nm thick Au layer (Au3) deposited on the PDMS grafted glass reveals densely packed, granular structures, whereas the 12 nm deposited Au (Au12) appears to have a more continuous morphology with interconnected domains. Upon annealing at 250 °C for 120 min, the Au3 restructures into compact, well‐separated nanodot arrays having a spherical shape. The 6 nm thick Au layer (Au6) forms larger, coarsened nanodots with increased interparticle spacing after annealing. Even though the shape and size are different, both Au3 and Au6 annealed samples exhibit dropwise nanodots. In contrast, the Au12 fails to fragment into individual nanodots but instead forms a fully connected morphology with internal voids. Solid‐state dewetting typically initiates along grain boundaries of the deposited metals.^[^ ^20^, ^22^ ^]^ Thinner metal layers provide a higher density of grain boundaries, thereby promoting the breakup of connected metal structure into isolated nanodots. Conversely, thicker metal layers tend to reorganize into more continuous or aggregated structures during annealing.

Optical properties of plasmonic metal nanostructures, governed by their localized surface plasmon resonance (LSPR), are strongly dependent on particle size, spatial distribution, and interparticle spacing.^[^ ^45^, ^46^, ^47^ ^]^ A visible color shift from bluish to goldish hues corresponds to changes in the LSPR of Au, which are specifically associated with increased nanodot size and enhanced interparticle coupling.^[^ ^48^ ^]^ Thus, morphological variations are directly reflected in the optical appearance of the films, as demonstrated by the color differences observed in the inset images of Figure 1b. UV–visible (UV–vis) spectroscopy provides a simple and effective tool to confirm these optical variations (Figure 1c). All as‐deposited metal layers exhibit broad absorption across the visible spectrum due to their irregular interconnected structures. After thermal annealing, these absorption bands become narrow, reflecting the transition to more discrete and uniform nanodot structures. The Au3 (blue line) displays a plasmonic peak centered at 539 nm with a full width at half maximum (FWHM) of 60 nm, consistent with the typical optical signature of ≈15 nm sized nanodots or nanoparticles.^[^ ^49^ ^]^ The annealed Au6 shows a broader peak centered at 593 nm with an FWHM of 105 nm, also indicating the formation of isolated nanodots after annealing. Here, the increased FWHM and redshifted peak center relative to the annealed Au3 suggest a larger average nanodot size and greater morphological irregularity. In case of the Au12, a broad absorption profile is retained even after annealing, which aligns with its continuous film‐like structure rather than the formation of discrete nanodots.

**Figure 2:**
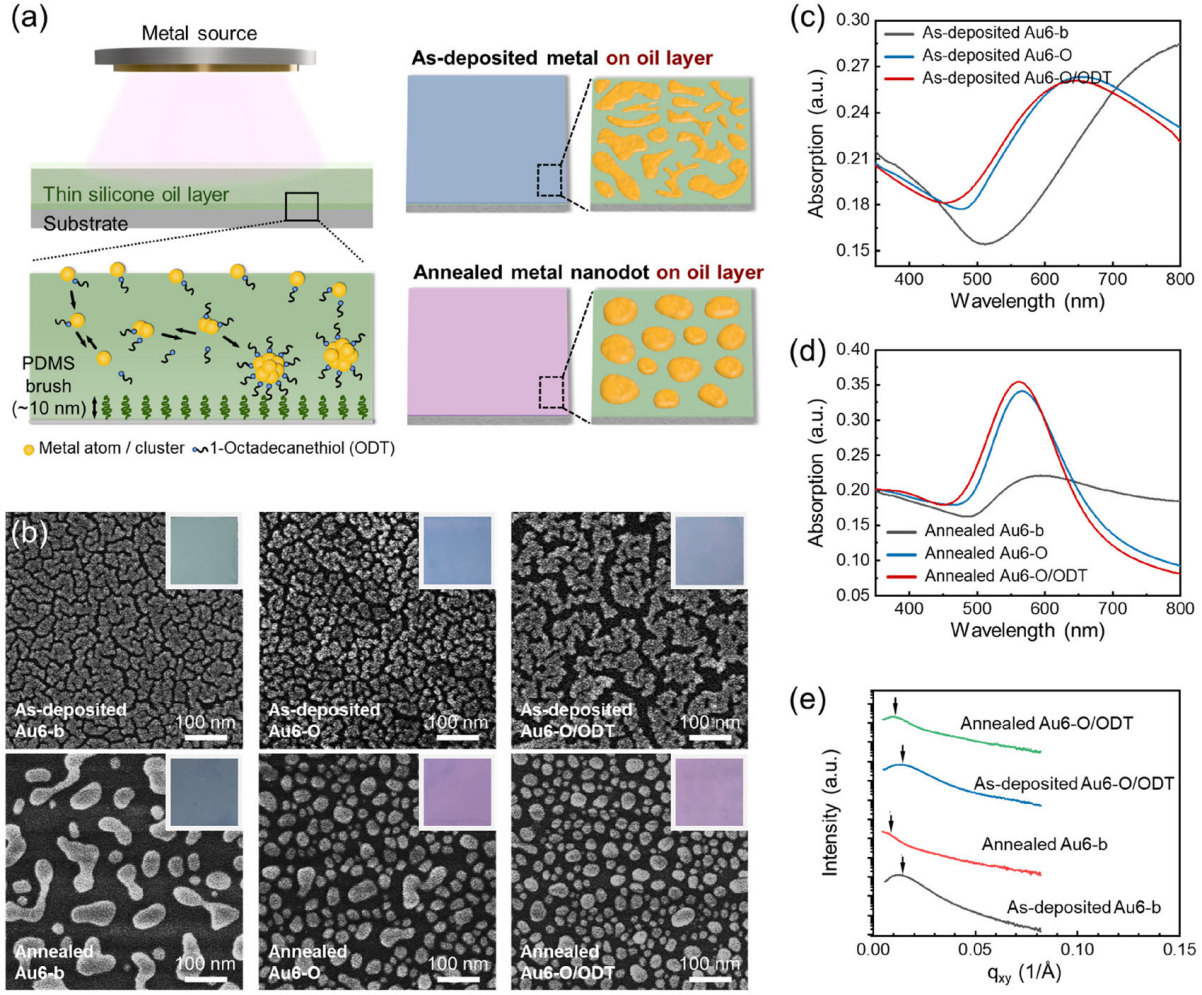
a) Schematic illustration of metal nanodot produced by physical deposition on thin silicone oil layer (the top‐left) and the stabilization mechanism of surfactant on metal nanodot array (the bottom‐left). The stabilized metal nanodot deposited onto thin oil layer at the as‐deposited and annealed state (right). b) Morphology differences of the 6 nm Au (Au6) on three different surfaces: PDMS grafted glass (Au6‐b), with silicone oil (viscosity: 100 cSt) (Au6‐O), and with oil containing ODT (Au‐O/ODT). Inset images are the corresponding optical image of substrates. UV–vis absorption spectra of (b). c) As‐deposited Au6 and d) annealed Au6. e) GISAXS curves of Au6 under different conditions, where the black (as‐deposited Au6‐b), red (annealed Au6‐b), blue (as‐deposited Au6‐O/ODT), and green (annealed Au6‐O/ODT) curves.

As shown above, solid‐state dewetting on bare solid substrates results in the formation of only small nanodots, with limited uniformity in size and shape. To fabricate nanodots with various sizes and more regular shapes, we apply a thin silicone oil layer onto the substrate, which helps to reduce the surface energy of the deposited metal. **Figure** **2**a schematically illustrates nanodot formation within the thin oil layer. In this study, silicone oil is applied onto the PDMS brush grafted glass by spin‐coating. Due to the interfacial compatibility and chemical similarity between the PDMS brush and silicone oil, a uniform thin oil layer is successfully formed and remains stable throughout the metal deposition process.^[^ ^44^, ^50^, ^51^ ^]^ In addition, the silicone oil can serve as a medium to contain a surfactant, 1‐octadecanethiol (ODT). To further stabilize the metal surface effectively, a thin oil layer containing ODT is applied to the substrate. Subsequently, metals are sputtered onto the substrates, followed by thermal annealing. Figure 2b presents scanning electron microscopy (SEM) images of the Au6 on three different substrate conditions: PDMS grafted glass 1) without oil (bare, ‐b), 2) with only oil (‐O), and 3) with oil containing ODT (‐O/ODT). Note that, to remove the oil layer prior to SEM imaging, we perform gentle hexane washing, which does not alter the nanodot morphology, as confirmed by UV–vis spectroscopy (Figure S1a,b, Supporting Information). Under more aggressive cleaning conditions, such as ultrasonication, partial damage is observed when the oil layer is present in the as‐deposited state; however, after annealing, the nanodots remain stable on the substrate (Figure S1c, Supporting Information). The top and bottom images represent the as‐deposited and annealed structures, respectively. Here, we primarily use an oil layer with a thickness of ≈60 nm. The thickness of the oil layer is determined by using atomic force microscopy (AFM) operated in force spectroscopy mode. Force–distance curves obtained during the tip approach are analyzed to quantify the oil thickness. As the AFM tip approaches the oil‐coated surface, the tip is wetted by the oil. As a result, capillary forces from the oil cause the cantilever deflection until the tip reaches the underlying hard substrate. The vertical distance between the initial deflection and the point of sharp deflection increase, which indicates substrate contact, is interpreted as the oil layer thickness (Figure S2, Supporting Information).^[^ ^52^, ^53^ ^]^ In addition, 250 °C is chosen as annealing temperature because a lower annealing temperature does not induce sufficient thermally driven solid‐state dewetting (Figure S3, Supporting Information). Thermally driven solid‐state dewetting is governed by two key thermodynamic factors: the Hüttig temperature (*T*_H_) and the Tammann temperature (*T*_T_).^[^ ^54^ ^]^ The *T*_H_, ≈0.3*T*_M_ (where *T*_M_ is the bulk melting point), corresponds to the onset of surface atom mobility, while *T*_T_ (≈0.5*T*_M_) denotes the temperature at which bulk atoms become mobile. Therefore, in principle, annealing above *T*_H_ can initiate dewetting accompanied by structural transformation. For Au (*T*_M_ = 1064 °C), *T*_H_ and *T*_T_ are 128 and 395 °C, respectively. However, as shown in (Figure S3a, Supporting Information), annealing at 150 °C activates surface diffusion but does not result in a homogeneous nanodot array. The Au6‐b exhibits filmwise dewetting without structural isolation by annealing. Although a higher annealing temperature might cause more effective dewetting, 250 °C is chosen to avoid thermal degradation of silicone oil, which occurs above 300 °C.

As shown in the images of the 6 nm deposited Au samples (top images of Figure 2b), the area covered by Au on the oil‐coated substrate (Au6‐O) is smaller than that on the bare surface (Au6‐b) and becomes smaller even on the oil with ODT (Au6‐O/ODT). After annealing, the structural differences become more pronounced (bottom images of Figure 2b). Compared to the Au6‐b, the Au6‐O/ODT yields smaller and more spherical nanodots. Mean local widths, obtained through quantitative shape analysis, are 17.5 ± 5.4 nm for Au6‐b and 12.3 ± 3.6 nm for Au6‐O/ODT (Figure S5, Supporting Information). For more details, see Supporting Note 2. These structural variations are corroborated by optical images (inset pictures in Figure 2b) and UV–vis spectroscopy, which complement beyond localized structural observations (Figure 2c,d). While all as‐deposited samples display broad LSPR spectra due to irregular film morphology, a blueshifted peak observed for the Au6‐O/ODT compared to the Au6‐b indicates a smaller mean nanodot size (Figure 2c). After annealing, both spectra exhibit features consistent with nanodot formation. The Au6‐O/ODT shows a peak centered at 562 nm with a FWHM of 92 nm, while the Au6‐b shows a peak centered at 593 nm with a FWHM of 105 nm (Figure 2d). This blueshift with reduced FWHM of the Au6‐O/ODT confirms smaller nanodot dimensions and improved size uniformity, attributed to the stabilizing effect of the ODT‐incorporated oil layer.

For further investigation of nanodot size tuning, we examine the dewetting behavior of metals with various deposition thicknesses, 3, 12, and 20 nm, in addition to 6 nm (Figure S6, Supporting Information). The Au3‐b and Au6‐b produce individual nanodots via solid‐state dewetting, even though their shapes and sizes are nonuniform. As the deposited metal volume increases, coalescence becomes more favorable than fragmentation into isolated nanodot features.^[^ ^33^ ^]^ Consequently, the Au12‐b and Au20‐b fail to undergo dewetting into discrete nanodots upon thermal annealing; instead, they form interconnected and aggregated domains with small embedded voids. On the other hand, the substrates coated with the O/ODT layer successfully produce nanodot arrays across all tested deposition thicknesses. Both SEM images and UV–vis spectra in (Figure S6, Supporting Information) show that larger nanodots are generated as the deposition thickness increases. In addition to the deposition thickness, the oil layer thickness also influences the size of the nanodots. We find that a thicker O/ODT layer results in the formation of smaller nanodots with more regular shapes with more blueshifted UV–vis spectra (Figure S7, Supporting Information), which is attributed to the extended stabilization time provided by the thicker oil layer before the metal contacts the underlying solid substrate.

To obtain statistically averaged structural information over a large sample area, in addition to UV–vis spectroscopy, grazing incidence small‐angle X‐ray scattering (GISAXS) measurements are performed at a constant incident angle of 0.50°. From the 2D GISAXS patterns (Figure S8, Supporting Information), 1D intensity profiles are extracted by line‐scanning along the *q*_xy_‐direction at a constant *q*_z_ value. These profiles are based on the reflection point in the 2D patterns for each sample and are presented as lines in Figure 2e: as‐deposited Au6‐b (black), annealed Au6‐b (red), as‐deposited Au6‐O/ODT (blue), and annealed Au6‐O/ODT (green). The as‐deposited samples, regardless of the presence of O/ODT, exhibit broad and weak scattering, indicating a semicontinuous morphology or a poorly defined particle arrangement. Upon annealing, the primary scattering peak (q*) shifts toward lower scattering vectors. The interparticle spacing (*d*) can be derived from q* values using the Bragg approximation, *d *= 2*π*/q*.^[^ ^55^ ^]^ In the absence of oil, q* decreases from 0.0129 to 0.0039 Å^−1^ upon annealing, corresponding to a 232% increase in *d*‐spacing from 487.8 to 1619.4 Å (Table S1, Supporting Information). Similarly, the Au6‐O/ODT also exhibits a shift in q* before and after annealing from 0.0135 to 0.0098 Å^−1^, corresponding to a 37% increase in *d*‐spacing, from 467.2 to 641.8 Å. These increases in *d*‐spacing verify successful solid‐state dewetting induced by thermal annealing.

**Figure 3:**
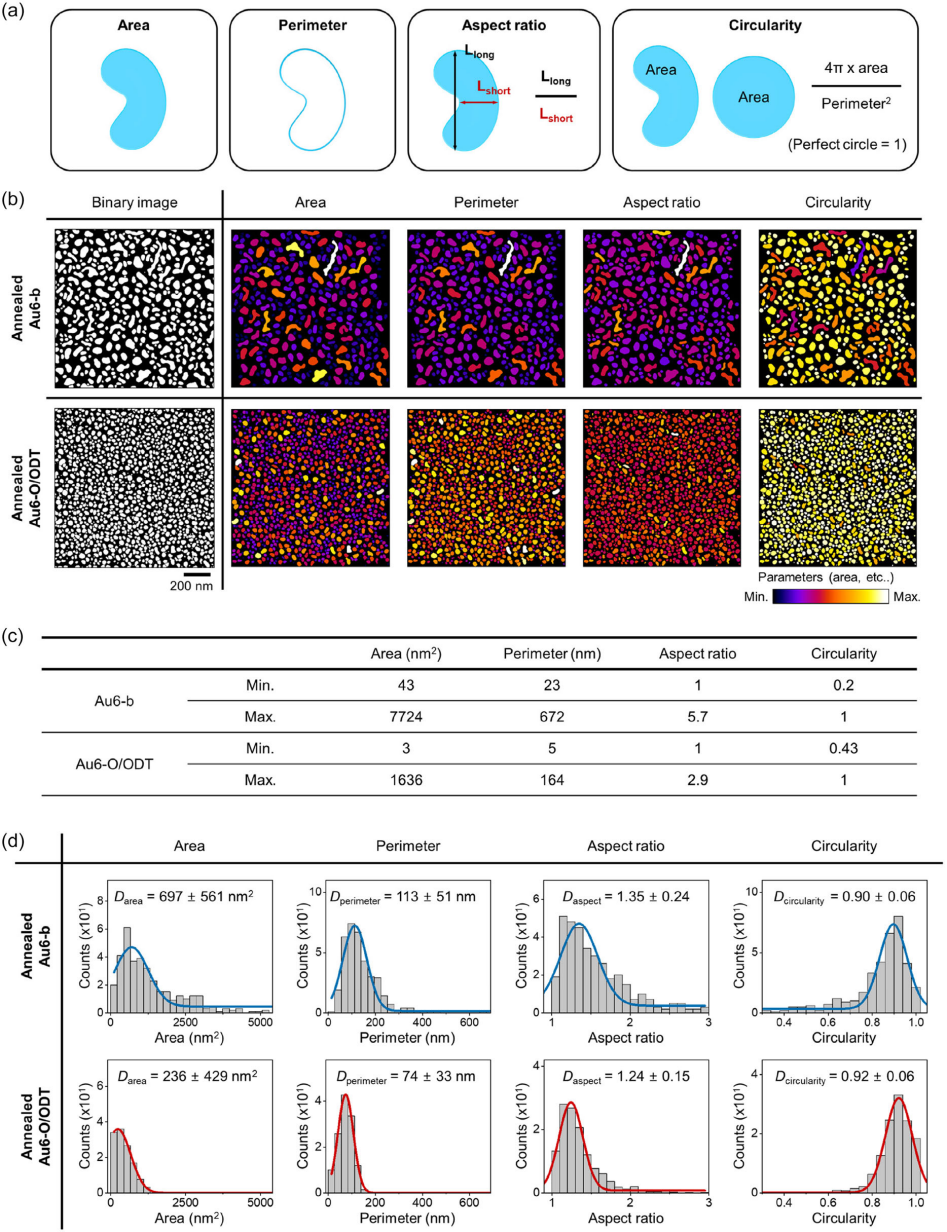
Morphological analysis of thermally dewetted Au nanodot array. a) Schematic representation describes the definition shape descriptors, including area, perimeter, aspect ratio (the longest length of particle divided by the shortest length of particle), and circularity (the projected area multiplying by 4*π* divided by the square of the perimeter). b) The converted image (binary image) obtained from SEM images and corresponding morphology maps color‐coded nanodots based on the defined variations in (a). c) Minimum (Min.) and maximum (Max.) value of color scales in (b). d) The variation of nanodot shape populations with Gaussian distribution curves. The values in the plot indicate the average values.

More quantitative parameters related to morphology are obtained through image analysis of the annealed nanodots. **Figure** **3** presents a detailed shape analysis of thermally dewetted Au nanodots. Several shape descriptors—including area, perimeter, aspect ratio, and circularity**—** are employed to characterize the geometric features of individual nanodots (Figure 3a). The morphology maps derived from SEM images are shown in Figure 3b. The grayscale image depicts the raw distribution of nanodots, where white regions represent Au and black regions indicate the background. The accompanying color‐coded maps display the nanodots according to their size and shape metrics. The color scale ranges in Figure 3b display the minimum and maximum values of each data to visualize the contrast of each nanodots (Figure 3c). Then, using color‐coded maps, statistical histograms fitted with Gaussian distribution curves are generated to quantify the variation of nanodot shape populations (Figure 3d). The most prominent change is a reduction in projected area. The average nanodot area decreases from ≈679 ± 561 nm^−2^ for Au6‐b to 236 ± 429 nm^−2^ for Au6‐O/ODT, suggesting that the surface of Au nanodots is effectively stabilized by the O/ODT layer. In parallel, the perimeter of the nanodots decreases from 113 ± 51 to 74 ± 33 nm. The difference in both the average values and the standard deviations indicates that the view that interfacial confinement restricts lateral growth, especially in the environment of the O/ODT. In addition, the aspect ratios, 1.35 ± 0.24 for the Au6‐b and 1.24 ± 0.15 for the Au6‐O/ODT, while the circularity increases from 0.90 ± 0.06 to 0.92 ± 0.06, showing that the nanodots become more isotropic and closer to a circular shape under O/ODT‐assisted conditions. These shape descriptors—aspect ratio and circularity—quantify the degree of anisotropy in nanodots and their deviation from symmetric configurations. A lower aspect ratio and higher circularity suggest that the nanodots undergo shape evolution toward more energetically favorable, spherical forms. This trend aligns with the fundamental behavior of metal nanostructures during thermal annealing, where surface energy minimization drives morphological transformation into more symmetric geometries. The O/ODT interfacial layer provides a low‐energy environment and simultaneously suppresses uncontrolled coalescence. Thus, the numerical differences in shape descriptors collectively highlight the role of the O/ODT layer in guiding isotropic nanodot formation driven by thermal dewetting.^[^ ^23^ ^]^

Nanodot arrays formed via solid‐state dewetting can also be achieved with other metals, such as silver (Ag) and copper (Cu). For example, Ag nanodots are created by Ag deposition followed by annealing at 250 °C (Figure S9, Supporting Information). Unlike the Au12‐b, even the Ag12‐b undergoes dewetting and produces isolated nanodots after annealing; the nanodots exhibit a mean local width ≈36 nm, even though the size is larger than those formed on Ag12‐O/ODT. These different dewetting behavior between Au and Ag may be caused by the lower *T*_H_ (≈97 °C) of Ag than Au (≈128 °C). Therefore, on the O/ODT‐coated substrate, nanodots with a mean local width of ≈9 nm are formed from 12 nm thick Ag films upon annealing, confirmed by SEM imaging and UV–vis absorption spectra.

**Figure 4:**
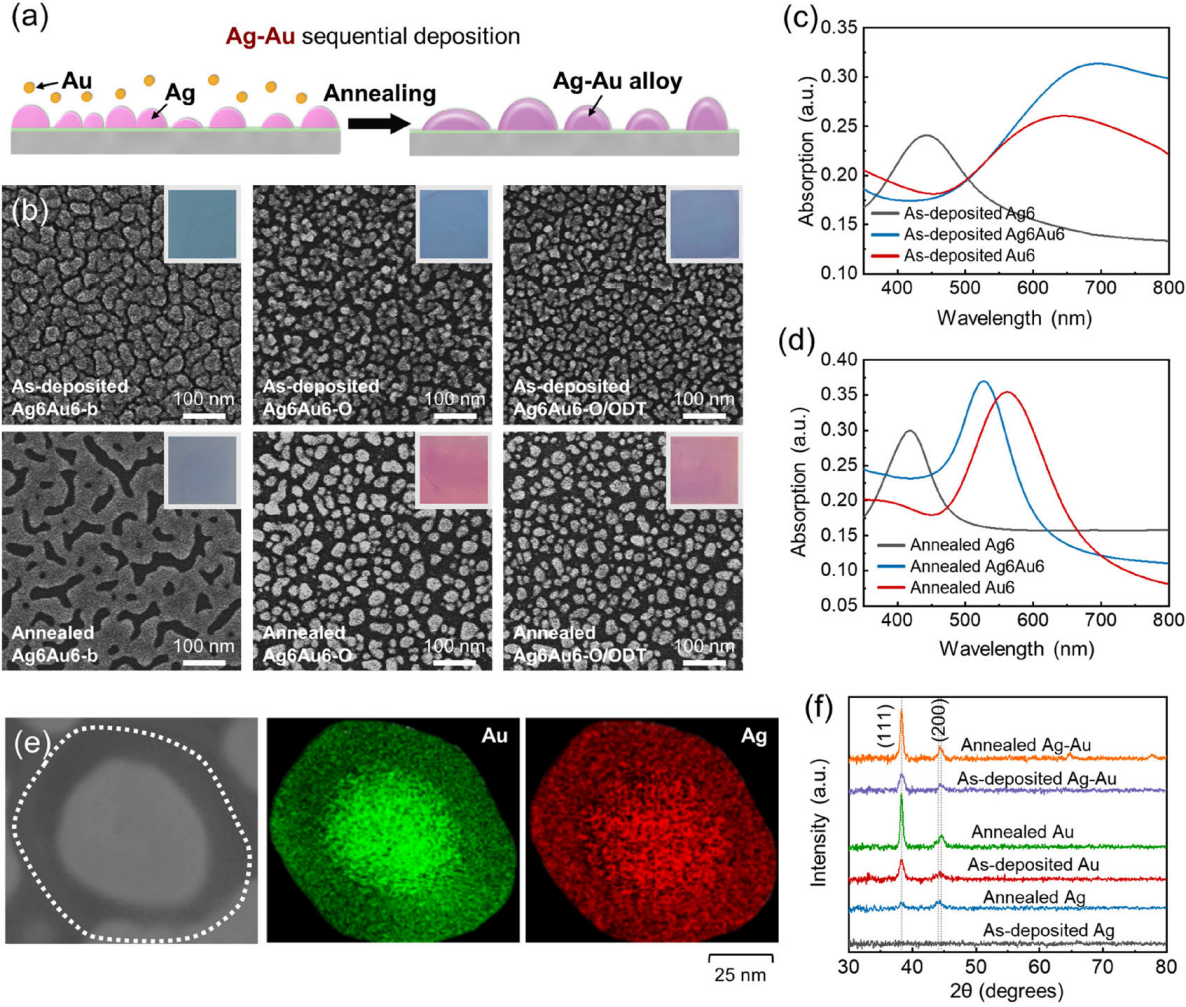
a) Schematic diagram of the sequential deposition and thermal annealing of Ag–Au. b) Structure of deposited Ag6Au6 (first layer: Ag, second layer: Au) on different surfaces. Inset images are the corresponding optical image of substrates. UV–vis absorption spectra. c) as‐deposited Ag6, Ag6Au6, and Au6. d) annealed Ag6, Ag6Au6, and Au6 on the O/ODT substrate. e) SEM based EDS mapping of thermally dewetted Ag6Au6‐O/ODT nanodot. f) XRD pattern of as‐deposited and annealed Ag, Au, and AgAu on the O/ODT surfaces.

Moreover, sequential metal deposition allows for the generation of bimetallic nanodots, such as Ag–Au alloy nanodots. To fabricate the Ag–Au nanodots, Ag is first deposited onto the substrate, followed by Au deposition (**Figure** **4**a). Subsequent thermal annealing successfully leads to the formation of alloy nanodots. Figure 4b shows the Ag–Au bimetallic structures formed with 6 nm deposition for each metal. Consistent with the tendency observed in single‐metal systems, the sequentially deposited bimetallic structures exhibit a well‐defined nanodot array with a narrow size distribution, particularly on the O/ODT‐coated substrate. The structural differences before and after annealing are verified by UV–vis spectra (Figure 4c,d). Before annealing, the LSPR peak of Ag6Au6‐O/ODT appears at a longer wavelength region compared to those of both Ag6‐O/ODT and Au6‐O/ODT. Interestingly, after thermal annealing, this spectrum shifts to a shorter wavelength, centered at 527 nm, which lies between the peaks of Ag6‐O/ODT and Au6‐O/ODT. This combined LSPR signal of the two metals, rather than individual resonances, suggests the formation of an alloy.^[^ ^56^, ^57^, ^58^ ^]^ Energy‐dispersive X‐ray spectroscopy (EDS) mapping acquired via SEM reveals that the Ag and Au signals are colocalized within the same nanodot (Figure 4e). In addition, X‐ray diffraction (XRD) patterns demonstrate structural changes in Ag, Au, and Ag–Au single and bimetallic nanodots induced by thermal annealing (Figure 4f). In the as‐deposited metal, no distinct diffraction peak corresponding to Ag is observed, probably due to the small amount of Ag on the substrate and/or its inherent polycrystallinity resulting from sputtering.^[^ ^59^, ^60^ ^]^ For Au, broad and weak peaks corresponding to the (111) and (200) orientations are detected. After annealing, both Ag and Au exhibit sharper diffraction peaks with increased intensity, indicating enhanced nanodot growth and improved crystallinity. In case of Ag–Au, the (111) plane peak appears at nearly the same position as those of pure Ag and Au (38.1°–38.2°). The (200) plane peak of Ag–Au is observed at ≈44.3°, which lies between the corresponding peaks of pure Ag and Au, confirming the formation of alloy nanodots. Importantly, since the PDMS polymer brush can be applied to a variety of substrate materials through surface functionalization, this strategy is not limited to glass alone.^[^ ^43^ ^]^ When combined with the formation of mono‐ and bimetallic nanodots via controlled deposition on diverse substrates, this system can be more effectively extended to applications, such as plasmonic, catalytic, and multifunctional nanodevice platforms.^[^ ^61^, ^62^, ^63^, ^64^ ^]^

**Figure 5:**
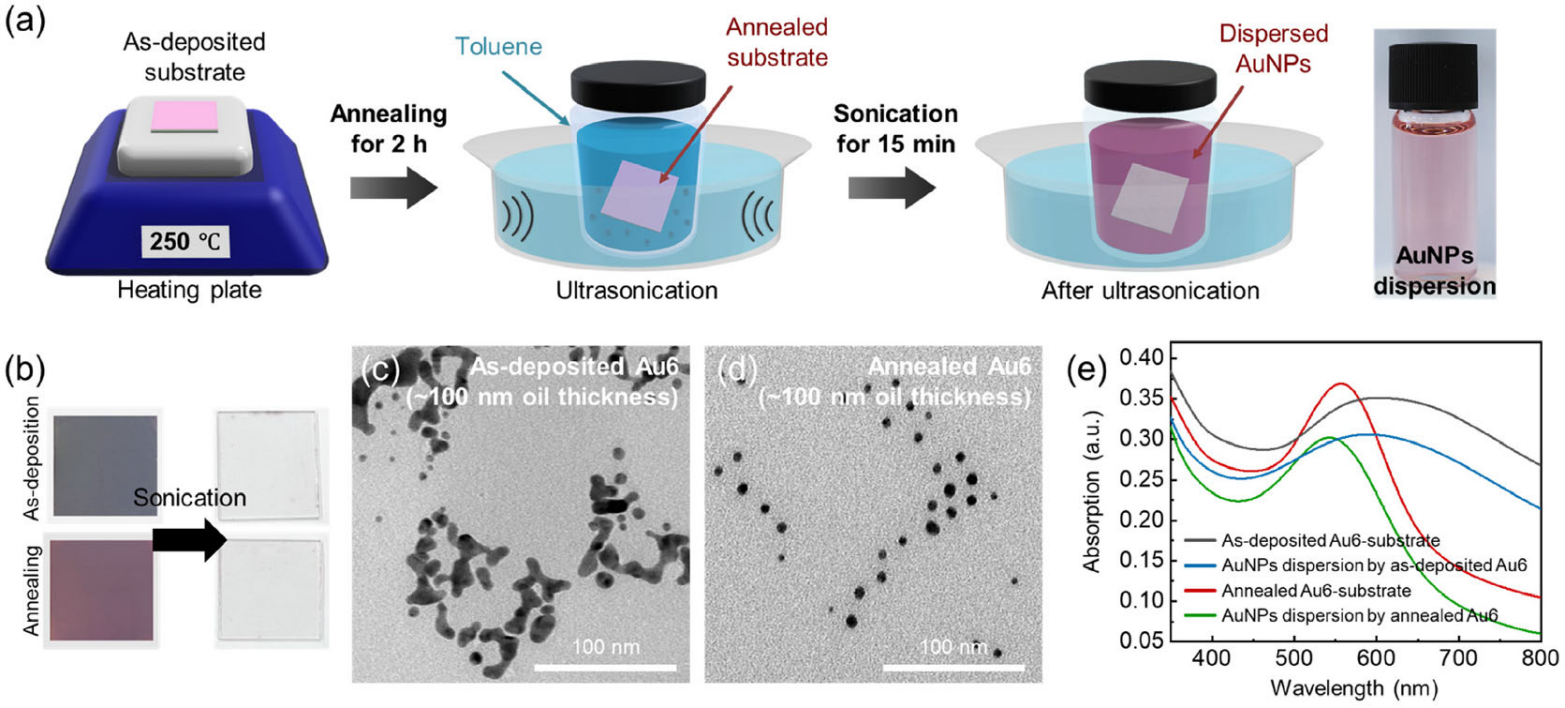
a) Schematic illustration of collection of AuNPs from the O/ODT‐coated substrates. b) Picture of the substrate with Au deposited on the O/ODT surface (≈100 nm) before and after sonication. TEM images of AuNPs collected on ≈100 nm oil layer substrate c) before annealing and d) after annealing. e) UV–vis absorption spectra of Au6 on oil layer of ≈100 nm substrate and AuNPs dispersion.

In addition to the formation of nanodot arrays on substrates, we also demonstrate a method for preparing colloidal nanoparticles by detaching nanodots from the substrate. As we discussed in (Figure S7, Supporting Information), smaller nanodots are generated when using a thicker oil layer. When the oil thickness exceeds ≈100 nm, the deposited Au can be readily detached from the substrate. It is expected that the Au nanodots become fully stabilized by ODT due to enough time before reaching the substrate through the thick O/ODT layer. The detachment process is schematically illustrated in **Figure** **5**a. To release the as‐prepared Au nanodots, the Au deposited O/ODT‐coated substrate is immersed in toluene, which dissolves silicone oil, followed by ultrasonication. As a result, Au nanodots are released into toluene as colloidal Au nanoparticles (AuNPs), accompanied by the O/ODT (Figure 5b). The right picture in Figure 5a shows the AuNP dispersion obtained by detaching of the annealed Au6‐O/ODT from the substrate. Even the as‐deposited Au6‐O/ODT, which exhibits a semicontinuous and irregular morphology, can be detached, as confirmed by transmission electron microscopy (TEM) (Figure 5c). In contrast, spherical AuNPs are obtained from the annealed Au6‐O/ODT, as shown in Figure 5d. The UV–vis spectrum of AuNP dispersion is nearly the same as that of the Au6‐O/ODT nanodot array on the substrate, with a minor shift of ≈9 nm attributed to differences in the surrounding medium (Figure 5e).^[^ ^65^, ^66^ ^]^ This result demonstrates that the AuNPs are dispersed into the solvent without significant changes in shape and size. In fact, nanodots formed on a ≈160 nm thick O/ODT layer can be detached even without sonication, suggesting that the Au nanodots are not strongly bound to the substrate under these conditions (Figure S12, Supporting Information). However, this also implies that nanodots formed within oil layers thicker than ≈160 nm are unsuitable for fixed‐array applications, thereby defining a practical upper limit for the oil layer thickness.

## 3 Conclusion

In this study, we propose a strategy for fabricating metal nanodot arrays on a solid substrate via thin oil layer‐assisted solid‐state dewetting. By depositing metals onto a silicone oil‐coated surface, followed by thermal annealing, continuous film‐like structures are transformed into isolated nanodot arrays. The addition of surfactants dissolved in the oil enables effective surface stabilization of the metal, thereby promoting solid‐state dewetting and resulting in improved size and shape uniformity of the nanodots. We investigate the effects of deposition thickness, oil layer thickness, and annealing temperature on dewetting behavior to achieve nanodots with tunable dimensions and morphologies. Furthermore, this method is readily extendable to bi‐ or multi‐metallic systems, producing alloy nanodots. This approach offers a simple, scalable, and lithography‐free route to construct nanodot arrays with tailored geometries and tunable functionalities. The ability to produce well‐defined nanodots with various compositions and states establishes a versatile platform for the design of metal nanodot arrays for applications in plasmonics, catalysis, photonics, and colloidal nanoparticle synthesis.

## 4 Experimental Section

4.1

4.1.1

##### Preparation of PDMS Grafted Glass & Thin Silicone Oil Coated Glass

PDMS grafted surface is prepared on a slide glass by following the literature.^[^ ^43^, ^44^ ^]^ The slide glass is sequentially washed with ethanol and acetone by ultrasonication for 10 min, respectively. Then, the washed glass is cleaned by oxygen plasma treatment (PDC‐002, Harrick, America) for 5 min. Subsequently, silicone oil (trimethylsiloxy‐terminated PDMS, viscosity: 100 cSt, MW = 5,970, Gelest Inc.) is applied to the substrate and heated at 100 °C for 48 h to graft a PDMS brush layer on the glass. Following the grafting reaction, residual oil is removed using toluene and tetrahydrofuran. To prepare thin silicone oil‐coated surfaces, the PDMS grafted glass is coated with silicone oil through spin‐coating at 4000 rpm for 60 s.

##### Oil Thickness Measurements

To fabricate oil‐coated substrates with varying thicknesses, silicone oil (viscosity: 100 cSt) is diluted in hexane to prepare solutions with concentrations of 0.1, 1, 2.5, and 5 wt%. These solutions are applied to the substrate to control the oil layer thickness. The resulting oil film thicknesses are characterized by using force spectroscopy in contact mode with an atomic force microscope (AFM) (JPK NanoWizard 4, Bruker, America). Prior to each measurement, the deflection sensitivity of the cantilever is calibrated by acquiring force–distance curves. For each sample, a grid of 5 × 5 force–distance measurements is recorded over the 100 μm × 100 μm area, with the scan speed set to 1 μm s^−1^. 10 samples are analyzed for each oil concentration to obtain average thickness values. The measured oil layer thicknesses are 11.2 ± 3.8, 61.9 ± 2.7, 99.4 ± 11.3, and 162 ± 7.3 nm for 0.1, 1, 2.5, and 5 wt% solutions, respectively. Additionally, 1 wt% of 1‐octadecanethiol (relative to the amount of silicone oil) is incorporated into the solutions as a surfactant.

##### Metal Deposition and Annealing

As‐deposited metals are prepared via sputtering deposition (MCS 010, BAL‐TEC, Germany) onto the PDMS grafted glass, either bare or coated with oil or oil/ODT layers. During sputtering, the vacuum pressure is maintained at 2 × 10^−2^ mbar, and the temperature is kept at 25 °C. The metal thickness is measured using the controller (QSG 070, BAL‐TEC, Germany). After deposition, the metal deposited substrates are placed on a heating plate and annealed at 250 °C under inert conditions.

##### Characterization of Metal Nanodot Array

During the annealing process, the plasmonic peak shifts of the annealed metals are monitored using ultraviolet–visible spectroscopy (UV–vis) (V‐770, JASCO, Japan). The surface morphologies are characterized by SEM (SIGMA 300, Carl Zeiss, Germany) and AFM. AFM imaging is performed using a cantilever with a resonance frequency of 300 kHz and a nominal spring constant of 26 N m^−1^. Furthermore, GISAXS is conducted using a Xenocs Xeuss 3.0 equipped with a 2D charge‐coupled device (CCD) detector (Eiger2 R, Dectris) under vacuum conditions. The incident X‐ray wavelength is *λ* = 1.54 Å. The detector is positioned at a distance of 1.1 m from the sample to collect 2D GISAXS patterns with an exposure time of 1200 s.

##### Characterization of Synthesized Metal Nanoparticles

For the deposited Au on substrates with oil layers over ≈100 nm, AuNPs can be detached by immersing the samples in toluene. The resulting NP dispersions are dropped onto copper grids and characterized by TEM (JEM‐F200, JEOL, Japan).

## Conflict of Interest

The authors declare no conflict of interest.

## Author Contributions

**Hyesun Hwang**: data curation (lead); formal analysis (lead); investigation (lead); visualization (lead); writing—original draft (equal); writing—review & editing (equal). **Jihye Kim**: formal analysis (supporting); investigation (supporting). **Seungbae Jeon**: formal analysis (lead); writing—original draft (supporting). **Seong‐Min Jo**: data curation (equal). **Sungmin Park**: formal analysis (equal); writing—original draft (supporting). **Hyosung An**: formal analysis (equal); visualization (equal). **Michael Kappl**: formal analysis (equal); writing—original draft (equal). **Hans‐Jürgen Butt**: investigation (equal); supervision (lead); writing—original draft (equal); writing—review & editing (equal). **Sanghyuk Wooh**: project administration (lead); writing—original draft (equal).

## Supporting information

Supplementary Material

## Data Availability

The data that support the ﬁndings of this study are available from thecorresponding author upon reasonable request.
