# Supplementary material for: Metal Nanodot Array via Thin Oil Layer‐Assisted Dropwise Solid‐State Dewetting
Source: Small Sci. 2025 Sep 11;5(11):2500311. doi: 10.1002/smsc.202500311 (PMC12622507; doi:10.1002/smsc.202500311)
Supplement: Supplementary file 1 — Supplementary Material [file SMSC-5-2500311-s001.pdf]

Supporting Information

Metal Nanodot Array via Thin Oil Layer-assisted Dropwise Solid-state Dewetting

Hyesun Hwang[1], Jihye Kim[1], Seungbae Jeon[2], Seong-Min Jo[3,4], Sungmin Park[2], Hyosung An[5], Michael Kappl[6], Hans-Jürgen Butt[6*], and Sanghyuk Wooh[1*]

[1]Department of Chemical Engineering, Chung-Ang University, 84 Heukseok-ro, Dongjak-ju,Seoul, 06974, Republic of Korea

[2]Advanced Materials Division, Korea Research Institute of Chemical Technology, 141 Gajeong-ro, Yuseong-gu, Daejeon 34114, Republic of Korea

[3]Department of Biomaterial Science, Pusan National University, 1268-50, Samrangjin-ro, Samrangjin-eup, Miryang-si, Gyeongsangnam-do, 50463, Republic of Korea

[4]Physical Chemistry of Polymers, Max Planck Institute for Polymer Research, Ackermannweg 10, 55128, Mainz, Germany

[5]Department of Petrochemical Materials Engineering, Chonnam National University, 19 Samdong 3-gil, Yeosu-si, Jeollanam-do, 59631, Republic of Korea

[6]Physics at Interfaces, Max Planck Institute for Polymer Research, Ackermannweg 10, 55128, Mainz, Germany

Keywords: Solid-state dewetting, Metal nanodot array, Metal deposition, Thin oil layer, Surface energy

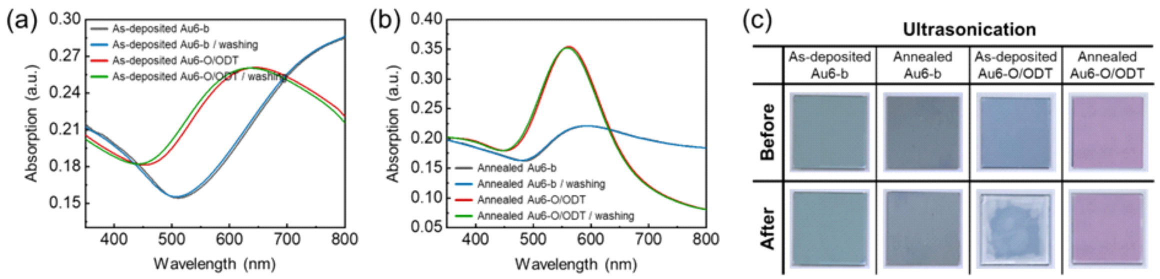

Figure S1. UV-vis absorption spectra of Au6 on ~60 nm thick oil coated substrate: (a) as-deposition and (b) after annealing. c) Pictures of the substrate of the Au6-b and Au6-O/ODT before and after ultrasonication treatment for 15 min.

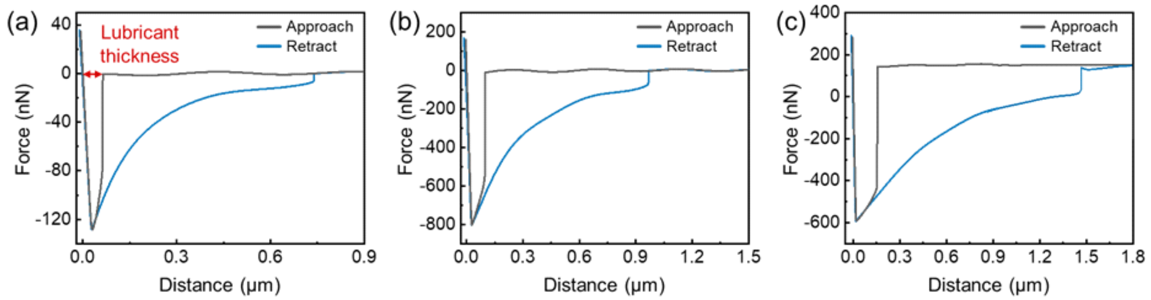

Figure S2. Representative force curves measured by the atomic force microscopy (AFM). Oil layer thickness formed from ODT-containing silicone oil solutions of varying concentrations in hexane. a) ~60 nm (silicone oil 1 wt% in hexane). b) ~ 100 nm (2.5 wt%). c) ~ 160 nm (5 wt%). The oil thickness increases with solution concentration after spin coating at 4000 rpm for 60 s.

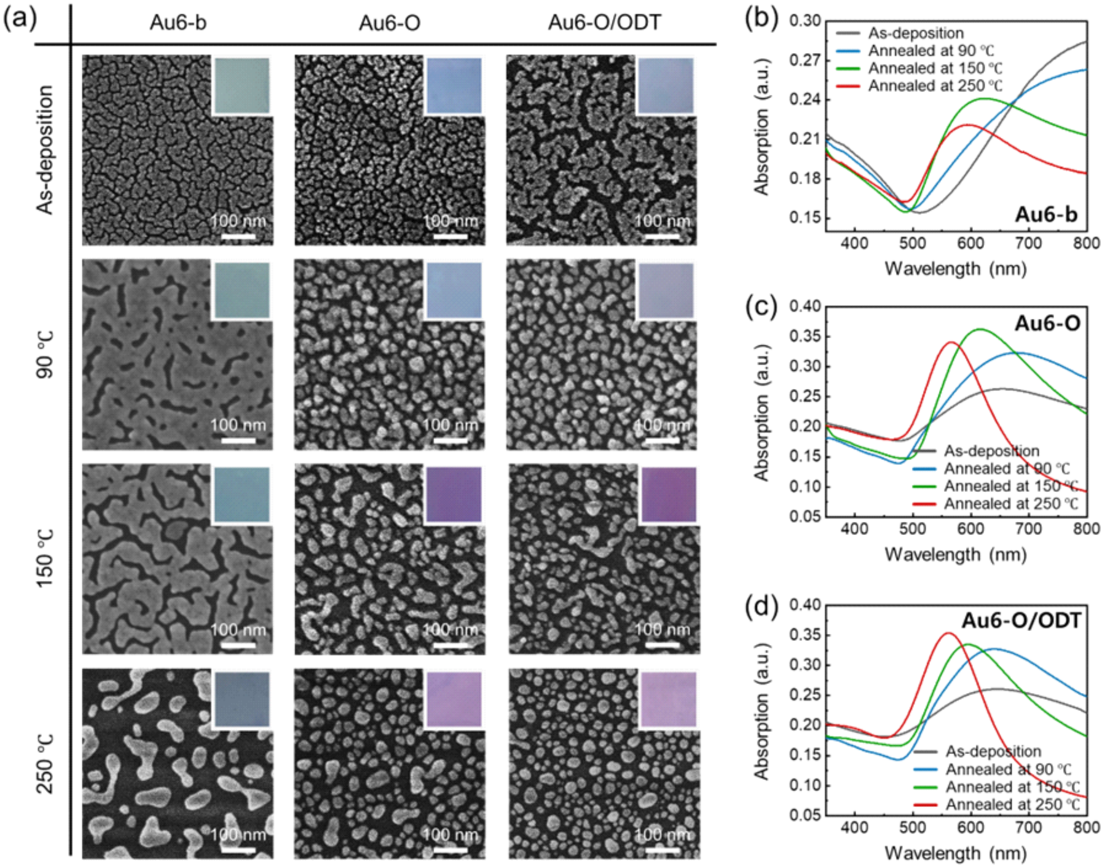

Figure S3. a) Morphologies of Au6 (thickness: 6 nm) of as-deposition, annealed at 90, ℃, 150 ℃, and 250 ℃. Inset pictures are the corresponding color of substrates. The annealing time is for 120 min. UV-vis absorption spectra of (b) Au6-b, (c) Au6-O, and (d) Au6-O/ODT depending on the annealing temperature.

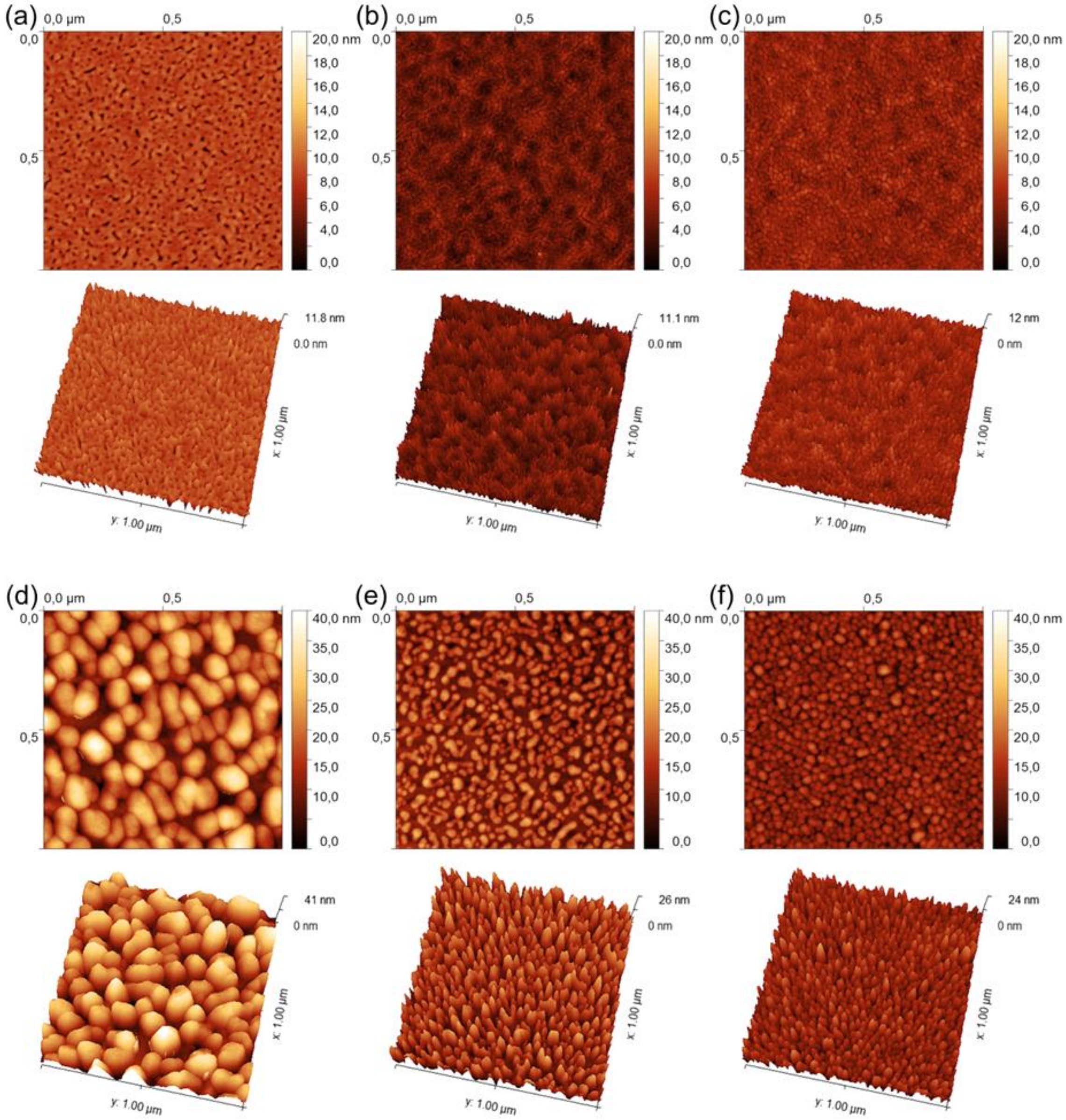

Figure S4. Atomic force microscopy (AFM) images. a) As-deposited Au6-b. b) As-deposited Au6-O. c) As-deposited Au6-O/ODT. d) Annealed Au6-b. e) Annealed Au6-O. f) annealed Au6-O/ODT. The annealing temperature is 250 ℃ for 120 min.

Supporting Note 1.

Morphological differences observed by atomic force microscopy (AFM) reveal that variations in interfacial conditions lead to distinct surface roughness and particle growth behavior (Figure S4). In the as-deposited state, the Au6-b exhibits a root mean square (RMS) roughness of 1.33 nm and an average height of 8.09 nm, indicating inhomogeneous particle formation due to uncontrolled aggregation of Au atoms on the bare substrate. In contrast, the Au6-O and Au6-O/ODT, in which the substrates are modified with silicone oil and oil/surfactant, display reduced RMS roughness values of 1.13 nm and 1.07 nm, and lower average heights of 4.21 nm and 5.91 nm, respectively. Upon annealing, the Au6-b shows a sharp increase in RMS roughness to 7.07 nm and in average height to 18.88 nm, indicating significant coalescence and vertical growth of particles due to unconfined diffusion. Meanwhile, the annealed Au6-O and Au6-O/ODT exhibit relatively moderate increases in roughness (4.88 nm and 3.43 nm) and average height (11.92 nm and 10.95 nm), respectively. These results demonstrate that the oil and surfactant layers serve as diffusion barriers, effectively regulating particle aggregation not only laterally but also vertically.

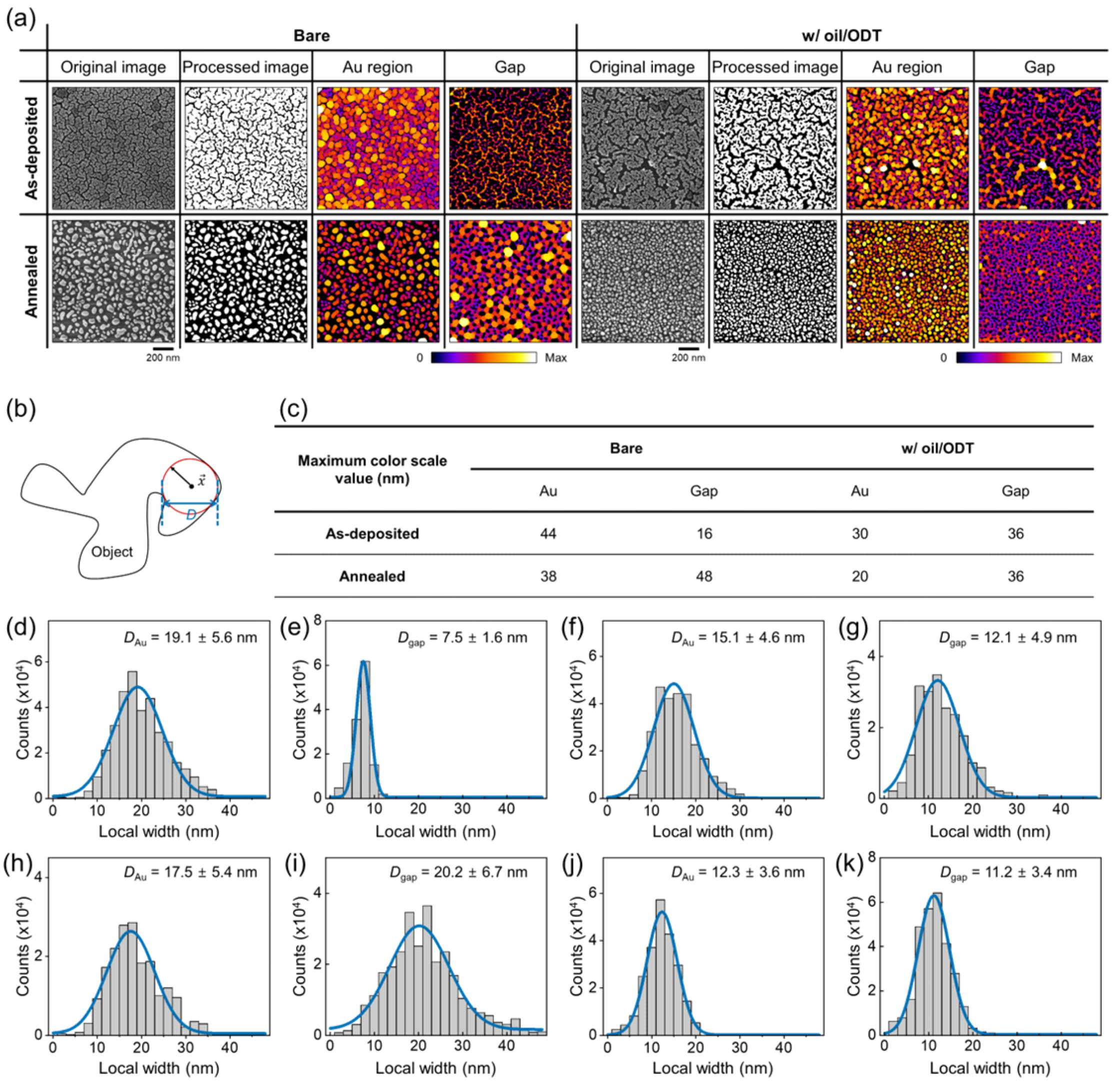

Figure S5. a) SEM images and corresponding binarized and color-coded map of as-deposited and annealed Au6-b and Au6-O/ODT. The color-coded map represents local width (*D*) of Au and gap region. b) The definition of local width is the diameter of the largest circle that fits inside the object. c) The maximum color scale values of the color maps of (a). d-k) Histograms and fitted gaussian distribution curves of local widths. d) Au, as-deposited Au6-b, e) gap, as-deposited Au6-b, f) Au, as-deposited Au6-O/ODT, g) gap, as-deposited Au6-O/ODT, h) Au, annealed Au6-b, i) gap, annealed Au6-b, j) Au, annealed Au6-O/ODT, and k) gap, annealed Au6-O/ODT. The values in the plot indicate the mean values.

Supporting Note 2.

To quantitatively assess the spatial morphology of Au nanodot arrays, we apply local thickness analysis to SEM images (Figure S5). This technique provides a spatially resolved measure of structural features by calculating the diameter of the largest circle that fits entirely within either Au or gap (void) domains (Figure S5b). Figure S5a presents the SEM images, binarized phase maps, and their corresponding color maps for Au6, comparing Au6-b and Aub-O/ODT, both in the as-deposited and annealed samples. Au and gap regions are represented by white and black regions in binary images, respectively. The scale in color-coded maps is adjusted to their individual maximum value in each dataset to give the best image contrast. These maximum values varied depending on the experimental condition, as summarized in Figure S5c. The value in Figure S5d-k represents the mean particle or gap width. In the as-deposited Au6-O/ODT, deposited metal exhibits reduced mean local width for Au domains (e.g., $D_{\mathrm{Au}} \approx 15.1$ nm) and larger spacing between Au (e.g., $D_{\mathrm{gap}} \approx 12.1$ nm), compared to as-deposited Au6-b ($D_{\mathrm{Au}} \approx 19.1$ nm, $D_{\mathrm{gap}} \approx 7.5$ nm). Upon thermal annealing, the difference between the two conditions is more significant. Annealed Au6-b shows particle growth and broader void distributions ($D_{\mathrm{Au}} \approx 17.5 \pm 5.4$ nm, $D_{\mathrm{gap}} \approx 20.2 \pm 6.7$ nm), showing uncontrolled coalescence and irregular nanodot formation. In contrast, annealed Au6-O/ODT maintains their particle distributions ($D_{\mathrm{Au}} \approx 12.3 \pm 3.6$ nm, $D_{\mathrm{gap}} \approx 11.2 \pm 3.4$ nm), corresponding to the formation of spatially uniform and well-confined nanodot. In addition, such narrowing in size and spacing distribution is directly linked to the spectral sharpening observed in UV-vis measurements, as shown in Figure 2c,d.

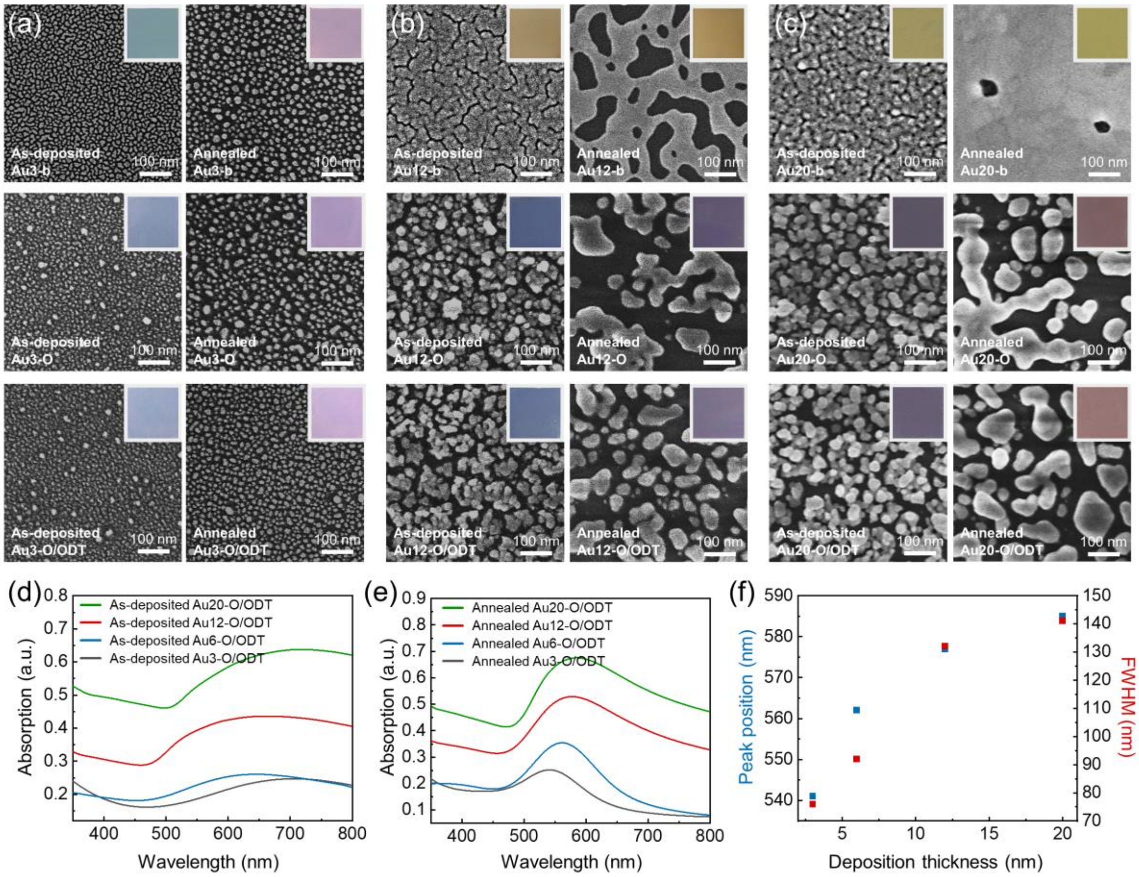

Figure S6. Au nanodot array as a function of Au thickness of a) 3 nm (Au3), b) 12 nm (Au12), and c) 20 nm (Au20), where bare (first row), with oil (second row), and with oil/ODT (third row). The left and right columns in (a-c) are as-deposited and annealed Au. Inset pictures in SEM are the corresponding optical images of substrate. The corresponding UV-Vis absorption spectra of d) as-deposited Au-O/ODT and e) annealed Au-O/ODT as a function of Au thickness. f) Plasmonic peak position and full width at half maximum (FWHM) of annealed Au-O/ODT.

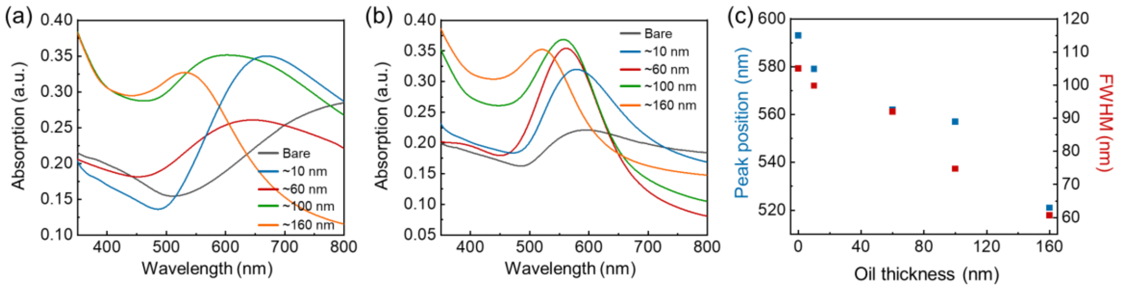

Figure S7. UV-vis absorption spectra of Au6-O/ODT depending on oil thickness (0, ~10, ~60, ~100, and ~160 nm). a) As-deposited Au6-O/ODT. b) Annealed Au6-O/ODT. c) Plasmonic peak position and full width at half maximum (FWHM) of annealed Au-O/ODT.

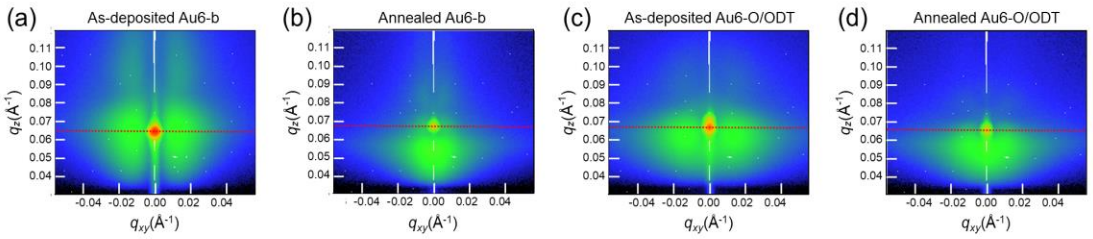

Figure S8. Two-dimensional grazing incidence small angle X-ray scattering (GISAXS) patterns of a) as-deposited Au6-b, b) annealed Au6-b, c) as-deposited Au6-O/ODT, and d) annealed Au6-O/ODT. The line cut denoted by red dotted line is performed by setting the q_z value based on the reflection point of each sample.

Table S1. q* peak shift before (as-deposited) and after annealing of 6 nm Au.

	**q* (Å^{-1})**	***d*-spacing (Å)**
As-deposited Au6-b	0.0129	487.8
Annealed Au6-b	0.0039	1619.4
As-deposited Au6-O/ODT	0.0135	467.2
Annealed Au6-O/ODT	0.0098	641.8

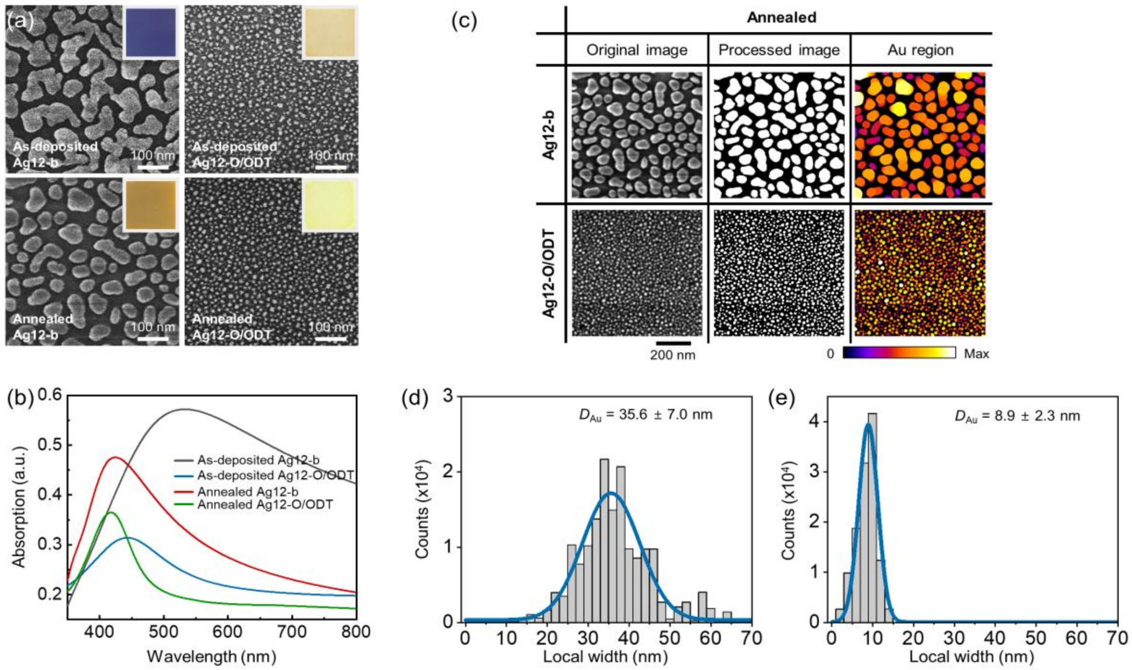

Figure S9. 12 nm silver (Ag12) deposition and annealing on bare and O/ODT substrates. Inset images are the corresponding optical images of substrates. UV-vis absorption spectra of b) as-deposited Ag12 and annealed Ag12. c) Color-coded map of local width of annealed Ag12-b and Ag12-O/ODT. Histograms and fitted gaussian distribution curves of local widths. d) Annealed Ag12-b. e) Annealed Ag12-O/ODT. The inset value in the plot represents the mean value.

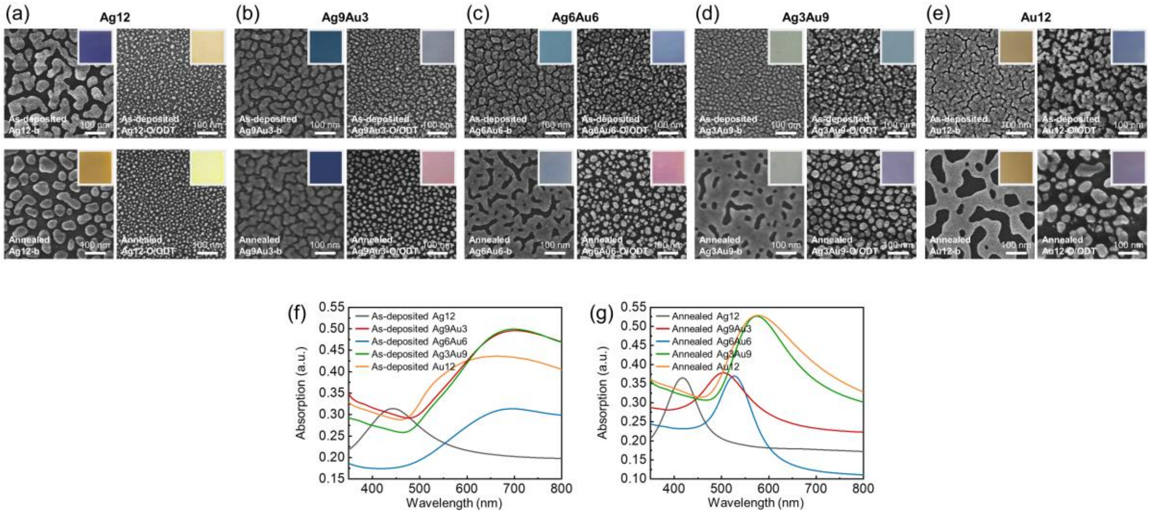

Figure S10. Sequentially deposited dropwise metal nanodot arrays with varying deposition ratio of (a) Ag 12 nm (Ag12), (b) Ag 9 nm-Au 3nm (Ag9Au3), (c) Ag 6 nm-Au 6 nm (Ag6Au6), (d) Ag 3 nm-Au 9 nm (Ag3Au9), and (e) Au 12 nm (Au12) on bare and O/ODT surfaces. Inset images are the corresponding optical images of substrates. UV-vis absorption spectra of (f) as-deposited and (g) annealed alloy arrays on O/ODT surfaces.

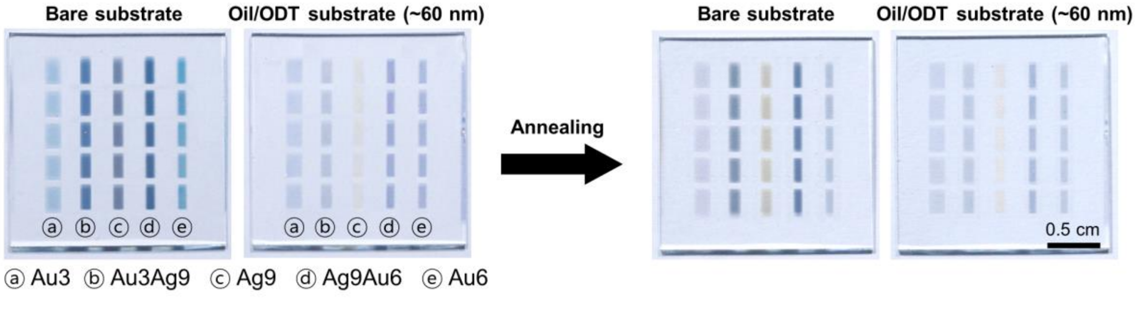

Figure S11. Patterned metal nanodot arrays on bare and O/ODT substrate before and after annealing.

Supporting Note 3.

Considering the total bimetallic film thickness of 12 nm, the bimetallic alloy structures are investigated by sequentially depositing Ag and Au with varying thickness: Ag 9 nm-Au 3 nm (Ag9Au3), Ag 6 nm-Au 6 nm (Ag6Au6), and Ag 3 nm-Au 9 nm (Ag3Au9) (Figure S10). All samples are annealed at 250 ℃. The size of the annealed alloy nanodots on the O/ODT-coated surface exhibits between those of pure Ag12 and Au12, with the LSPR peak shifting from blue to red as the Au content increases. The morphology also evolves from smaller and more spherical nanodots (in Ag-rich compositions) to gradually larger and more irregular shapes (in Au-rich compositions). Furthermore, by combining patterned masks during metal deposition with variations in film thickness, deposition sequence, metal composition, and other processing parameters, it is possible to fabricate metal nanodot arrays with diverse morphologies and optical properties (Figure S11).

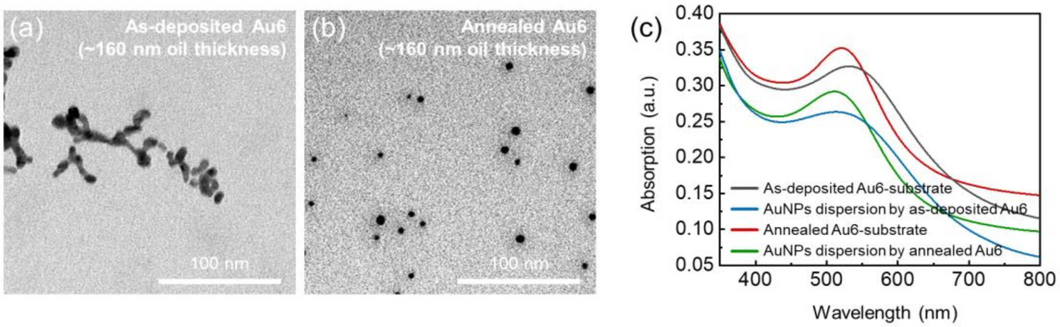

Figure S12. AuNPs collected on ~160 nm thickness oil layer substrate of a) as-deposition and b) after annealing. c) UV-vis absorption spectra of Au6 on oil layer of ~160 nm and after detachment from the substrate as a dispersion.
